# The speed of our mental soundtracks: Tracking the tempo of involuntary musical imagery in everyday life

**DOI:** 10.3758/s13421-015-0531-5

**Published:** 2015-06-30

**Authors:** Kelly Jakubowski, Nicolas Farrugia, Andrea R. Halpern, Sathish K. Sankarpandi, Lauren Stewart

**Affiliations:** Department of Psychology, Goldsmiths, University of London, New Cross Road, New Cross, London, SE14 6NW UK; Department of Psychology, Bucknell University, Lewisburg, PA USA; School of Nursing and Health Sciences, University of Dundee, Dundee, UK

**Keywords:** Music cognition, Imagery, Involuntary musical imagery, Involuntary memory, Spontaneous cognition, Tempo

## Abstract

The study of spontaneous and everyday cognitions is an area of rapidly growing interest. One of the most ubiquitous forms of spontaneous cognition is involuntary musical imagery (INMI), the involuntarily retrieved and repetitive mental replay of music. The present study introduced a novel method for capturing temporal features of INMI within a naturalistic setting. This method allowed for the investigation of two questions of interest to INMI researchers in a more objective way than previously possible, concerning (1) the precision of memory representations within INMI and (2) the interactions between INMI and concurrent affective state. Over the course of 4 days, INMI tempo was measured by asking participants to tap to the beat of their INMI with a wrist-worn accelerometer. Participants documented additional details regarding their INMI in a diary. Overall, the tempo of music within INMI was recalled from long-term memory in a highly veridical form, although with a regression to the mean for recalled tempo that parallels previous findings on voluntary musical imagery. A significant positive relationship was found between INMI tempo and subjective arousal, suggesting that INMI interacts with concurrent mood in a similar manner to perceived music. The results suggest several parallels between INMI and voluntary imagery, music perceptual processes, and other types of involuntary memories.

Empirical investigations of both non-volitional cognition and everyday thought processes have historically been neglected, due in part to the difficulty of harnessing these mental activities within a laboratory setting (Smallwood & Schooler, 2006, 2015). However, these mental processes comprise a substantial proportion of human cognition (McVay, Kane, & Kwapil, 2009) and provide an avenue for highly ecological research that can complement and extend traditional laboratory-based approaches. In recent years, the implementation of novel research designs has allowed researchers to begin to gain an understanding of spontaneous and naturalistic cognitions, including involuntary memories (e.g., Berntsen, Staugaard, & Sørensen, 2013), mind wandering (e.g., Killingsworth & Gilbert, 2010), and everyday thoughts within naturalistic settings (e.g., Hektner, Schmidt, & Csikszentmihalyi, 2006). The present study aims to further advance this area of research by introducing a new method for studying a type of everyday cognition related to music.

Involuntary musical imagery (INMI, or “earworms”) is the experience of a section of music coming into one’s mind involuntarily – without any intention to retrieve or recall the music – that immediately repeats at least once, without conscious effort to replay the music. Thus, INMI is characterized by two primary features: (1) it is recalled via associative and unplanned retrieval mechanisms, and (2) it is involuntarily repetitive in nature. These two characteristics serve to distinguish INMI from other related musical cognitions such as voluntary musical imagery, which is imagined music that is strategically retrieved (e.g., Zatorre & Halpern, 2005), musical “mind pops,” which comprise brief, single spontaneous appearances of a tune in the mind without repetition (e.g., Kvavilashvili & Anthony, 2012), and musical hallucinations, which are mental representations of musical sounds that are misattributed as originating from the external environment (e.g., Griffiths, 2000). The unplanned nature of retrieval from memory that is implicated in INMI also suggests some parallels between INMI and other types of involuntary memories. Indeed, INMI has been classified by some researchers as a type of involuntary semantic memory (Kvavilashvili & Mandler, 2004). However, INMI diverges from other involuntary memories in its high degree of repetitiveness, which is uncommon in most other involuntary memories. It has been suggested that this repetitiveness within INMI may stem from an exaggeration in the mind of the already highly repetitive nature of much of the music that exists in the Western world (Margulis, 2014).

INMI is a regularly occurring and widespread phenomenon in Western society. In a large-scale online survey, approximately 90 % of respondents reported that they experienced INMI at least once per week (Liikkanen, 2012). INMI has been reported in two studies as the most common type of involuntary semantic memory (Kvavilashvili & Mandler, 2004; Liikkanen, 2012), and reports of INMI experiences have been gathered from many countries across the globe (Liikkanen, Jakubowski, & Toivanen, in press). As such, INMI presents valuable opportunities to investigate everyday, spontaneous cognition.

## Previous involuntary musical imagery (INMI) research

INMI generally comprises the repetitive looping of short fragments of music, rather than whole songs (Brown, 2006; Liikkanen, 2012), and is often subjectively reported to be an authentic mental replication of the musical content of the original tune (Brown, 2006; Williamson & Jilka, 2013). INMI episodes reported in one diary study ranged widely in duration, from 2 to 240 min, with a median duration of 36 min (Halpern & Bartlett, 2011). Another diary study reported a mean INMI episode duration of 27.25 min (Beaman & Williams, 2010). INMI can occur for a wide range of genres of music, including pop, rock, classical, children’s songs, TV jingles, and film music (Beaman & Williams, 2010; Halpern & Bartlett, 2011; Hyman et al., 2013).

INMI often coincides with states of diffused attention, occurring frequently during housework, walking, or other routine tasks (Floridou & Müllensiefen, 2015; Hyman et al., 2013; Williamson et al., 2012), which is a similarity to other types of involuntary memories (Berntsen, 1998; Kvavilashvili & Mandler, 2004). In terms of evaluations of the experience, INMI is more often rated as emotionally positive or neutral than negative (Beaman & Williams, 2010; Halpern & Bartlett, 2011; Liikkanen, 2012). When INMI episodes do become troublesome or worrying, many people engage in both active and passive coping behaviors in an attempt to eradicate the unwanted INMI (Beaman & Williams, 2010; Williamson, Liikkanen, Jakubowski, & Stewart, 2014). Studies on individual differences have revealed links between INMI propensity and/or duration of INMI episodes and openness to experience, neuroticism, transliminality, schizotypy, obsessive-compulsive traits, and musical engagement (Beaman & Williams, 2013; Beaty et al., 2013; Floridou, Williamson, & Müllensiefen, 2012; Müllensiefen, et al., 2014; Wammes & Barušs, 2009).

One limitation of previous INMI research is that much of this evidence is based primarily on subjective reports regarding the inner experience of music. For instance, participants in some studies have verbally reported that INMI often represents a highly authentic mental replication of a familiar song (Brown, 2006; Williamson & Jilka, 2013), but researchers have not investigated the degree of precision with which the imagery replicates the original music in terms of pitch, tempo, lyrics, etc. The present study implemented a new method that quantitatively measured one musical feature of INMI – tempo – as it occurred during daily life by asking participants to tap to the beat of their INMI while wearing a wrist-worn accelerometer. By obtaining tempo information for individual INMI episodes, the present study was able to gain detailed insights into several questions of interest to researchers of INMI in a more objective manner than has previously been possible. Specifically, the research questions investigated in the present study concerned (1) the precision of memory representations within INMI, and (2) the interactions between INMI and concurrent affective state.

## Investigating the precision of INMI tempo recall

The precision of *deliberately recalled* musical memories has previously been investigated in laboratory-based studies. These studies have suggested that the pitch and tempo of familiar music are generally recalled (1) highly veridically, in comparison to an original, standard version of a song (Frieler et al., 2013; Levitin, 1994; Jakubowski, Farrugia, & Stewart, 2014; Levitin & Cook, 1996) and (2) highly consistently, in multiple trials across single participants (Halpern, 1988, 1989). In a study of voluntary musical imagery for tempo, Halpern (1988) reported a significant correlation between the tempo at which participants set familiar tunes in a perceived music condition and the tempo at which they imagined the same tunes in an imagery condition. However, she also found evidence for a “regression to the mean” for imagined tempo, such that relatively slow songs tended to be imagined faster than their preferred perceived tempo, and relatively fast songs tended to be imagined slower than their preferred perceived tempo.

In the present study, the veridicality of *involuntarily recalled*, everyday occurrences of musical imagery was investigated for songs that exist in canonical (standard, recorded) versions by comparing INMI tempo measurements to the tempo of the original songs. Veridical representations of musical tempo within INMI would suggest a parallel between the memory mechanisms implemented in deliberately recalled music and spontaneous musical imagery occurring within a naturalistic setting. Such a finding would also provide links to other types of involuntary memory, as involuntary autobiographical memories have often been found to be as, or even more, specific and detailed in comparison to voluntary autobiographical memories (Berntsen, 1998; Mace, 2006; Schlagman & Kvavilashvili, 2008). However, if temporal information was not preserved with high veridicality during INMI, this could suggest that other elements, such as affective state, might play a role in influencing the stability of tempo within INMI. The present design also allowed for the investigation of two secondary questions regarding temporal veridicality: (1) the influence of recent hearing of a song on veridicality of tempo recall, and (2) whether evidence for a regression to the mean could be found for INMI, similar to that reported for voluntary imagery by Halpern (1988).

The temporal consistency between multiple INMI episodes of the same song was explored in the present research by analyzing the tempi of songs that were repeatedly experienced as INMI within the same participant over the data collection period. Work by Byron and Fowles (2013) has shown a quick exponential decay in the recurrence of the same song as INMI, thus the number of instances of recurrent INMI songs was expected to be few. Nevertheless, this exploratory analysis contributed to our overall investigation into the temporal stability of the INMI experience.

## Investigating the relationship between INMI and affective states

If spontaneously recalled musical memories are found to rely on similar memory mechanisms to deliberately retrieved music, might they also serve similar functions to purposeful music selection? One of the most common uses of music within Western society is for mood regulation (Juslin & Laukka, 2004; Saarikallio & Erkkilä, 2007; Sloboda, O’Neill, & Ivaldi, 2001; Tarrant, North, & Hargreaves, 2000), and a handful of existing studies provide support for the idea that imagined music might also serve as a mood regulatory mechanism in the absence of an external music source. Participants in qualitative research have reported associations between their current mood and the type of INMI they experience (Williamson et al., 2012), and diary-based methods have revealed that INMI are more frequent in more alert mood states (Bailes, 2012). Research on voluntary musical imagery has revealed parallels between the decoding of emotion in music perception and imagery (Lucas, Schubert, & Halpern, 2010), indicating that music can convey similar emotions whether it is imagined or heard aloud. Additionally, research from the involuntary autobiographical memory literature indicates that these types of memories are often more emotional than their deliberately recalled counterparts, suggesting that involuntary retrieval of memories might even enhance their emotional qualities (Berntsen & Hall, 2004). However, no previous evidence exists as to whether certain musical dimensions of the INMI experience might relate to specific mood constructs, in a similar fashion to the way in which different features, such as the tempo, musical mode, or texture, of a piece of music can elicit different emotional responses during music listening (Husain, Thompson, & Schellenberg, 2002; Webster & Weir, 2005). As such, the second main aim of the study was to use our newly developed measures for capturing the tempo of INMI in order to investigate how musical features of the INMI experience might relate to one’s concurrent affective state.

Hypotheses for this research question were based on previous findings regarding the relationships between features of perceived music and emotional response. Several previous studies have revealed a link between musical tempo and arousal. Listening to fast tempo music can increase subjective arousal (Husain et al., 2002), fast tempo music is preferred in high arousal conditions such as exercise (Edworthy & Waring, 2006; North & Hargreaves, 2000), and physiological arousal can increase tempo judgments when participants are asked to indicate the tempo that “sounds right” for familiar, non-canonical songs (Jakubowski, Halpern, Grierson, & Stewart, 2015). As such, a positive relationship was predicted between subjective arousal and INMI tempo. Emotional valence appears to be less clearly related to musical tempo, but has been related to other features of music such as musical mode, i.e., major versus minor, such that the major mode is associated with positive emotional valence and the minor mode with negative valence (Gagnon & Peretz, 2003; Husain et al., 2002; Webster & Weir, 2005). In accordance with this previous research, the musical mode for each reported INMI song was also determined, with the prediction that major mode INMI would co-occur with more positive emotions than minor mode INMI.

## Summary of research questions

In summary, the present study employed a novel method to collect INMI tempo data during participants’ everyday activities over the course of 4 days. The acquired data were used to investigate two specific research questions. The first main question examined the precision of tempo recall within INMI, specifically in regard to veridicality and consistency. Evidence for veridical and consistent recall of the tempo of INMI would provide parallels to previous findings on deliberately recalled music (e.g., Halpern, 1988; Levitin & Cook, 1996). The second main question examined the relationships between musical features of INMI, specifically tempo and musical mode, and self-reported affective states, specifically in terms of arousal and valence. Evidence for relationships between INMI tempo and concurrent arousal and between INMI mode and emotional valence would provide parallels to previous findings on music listening (e.g., Edworthy & Waring, 2006; Husain et al., 2002). The results of the study represent a first step towards an understanding of temporal aspects of INMI within daily life.

## Method

### Design

A naturalistic study was conducted in which participants (1) tapped to the beat of their INMI while wearing an accelerometer that recorded their movements and (2) recorded information about each INMI episode in a diary during their daily lives over a period of 4 days.

### Participants

Participants were 17 volunteers (seven male), aged 20 to 34 years (*M* = 24.59, *SD* = 4.20). All participants were recruited on the basis that they reported experiencing earworms^1^ several times a day and were screened in advance in order to exclude any prospective participants who exhibited difficulties in tapping to the beat of musical imagery. The screening task involved tapping to the beat of familiar, voluntarily imagined songs in the laboratory. Any participant who was not an outlier^2^ on this task in terms of tapping variability was deemed eligible for inclusion in the study.

The sample comprised both musically trained and untrained participants. All participants received modest monetary compensation.

### Ethics statement

The study protocol was approved by the ethics committee of Goldsmiths, University of London, UK. Written informed consent was obtained from all participants.

### Materials

#### Measuring INMI tempo

To record INMI tempo, a GeneActiv wrist-worn accelerometer was employed.^3^ This device resembles a wristwatch and is a noninvasive tool for measuring participants’ movement data throughout the day (Rowlands et al., 2014; Zhang, Rowlands, Murray, & Hurst, 2012). Measurements for the present study were taken at the GeneActiv’s maximum sampling rate of 100 Hz. This maximum sampling rate imposed some limitations on the device’s ability to measure very fast tapping speeds. However, at the mean tempo of 100.9 beats per minutes (bpm) reported for INMI episodes in the present study the degree of uncertainty in the accelerometer measurement was only 1.6 bpm. Participants were asked to tap to the beat each time an earworm occurred while wearing the device.

In order to validate the viability of the GeneActiv device for measuring tapping data, a pilot study was conducted in which self-paced tapping (slow, medium, and fast tempi) was simultaneously measured by tapping on a laptop touchpad while wearing the accelerometer. Timing of tap onsets were registered by the laptop touchpad using MAX 6.1,^4^ and were also extracted from the accelerometer data using the analysis procedure described below (see *Tapping Data Analysis*). Both tap onset time series were processed to calculate the tempo of the series in bpm. All tempi calculated with the accelerometer data were within 1 bpm of the tempi measured using the touchpad.

#### Self-report diary measures

A paper diary was given to participants for reporting on the occurrence and circumstances of each earworm experienced over the 4-day period. Each page comprised 11 questions pertaining to a single earworm episode; participants were asked to fill in this booklet once they had finished tapping to the beat of the earworm. Each page of the diary asked for the time and date of the episode, the time the diary questions were completed, the name, artist, and section of the earworm song, the last time the song was heard aloud, whether the episode occurred during a repetitive movement (e.g., walking/running), and information on internal or external events that might have triggered the earworm episode (see Appendix 1 for full set of questions). The categories for the question on how the earworm episodes were triggered were based on previous large-scale, qualitative research (Williamson et al., 2012). The diary also comprised seven mood pairs used in previous musical imagery research by Bailes (2006, 2007, 2012) that were adapted from a study of music in everyday life by Sloboda et al. (2001). These seven mood pairs group into three factors: Positivity, Arousal, and Present Mindedness. Participants were asked to rate their mood on these seven scales in terms of the way they felt just before the earworm began.

### Procedure

**Fig. 1:**
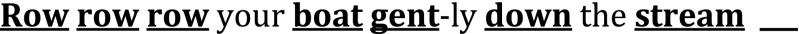
Example text used to explain the meaning of a “beat” to participants. Bold and underlined syllables correspond to beats in the music

Participants were asked to choose a 4-day block of time during which they felt they could complete the study most effectively. Each participant then met with the experimenter for approximately 15 min to receive the instructions and materials for the study. The experimenter provided a definition for the term “earworm,” as follows: “An earworm is a short section of music that comes into your mind without effort (it is involuntary; without any intention to retrieve or recall the music) and then repeats by itself (immediately repeated at least once, on a loop, without you consciously trying to replay the music).” Participants were instructed that whenever they experienced an earworm during the next 4 days, they were to tap along to the beat of the music as closely as possible to what they heard in their head while wearing the accelerometer device. They were asked to tap at least 20 times during each earworm episode. Examples of familiar songs (“Jingle Bells” and “Row, Row, Row Your Boat”) were provided to ensure that participants understood what was meant by the beat of the music (see Fig. 1). The experimenter then demonstrated the tapping method, in which participants were asked to tap with their full forearm on their leg. The experimenter also showed the participants how to press the button on the accelerometer to serve as a marker of the end of each tapping episode. No button press was required at the start of the tapping episode so that participants could begin tapping as soon as they noticed an earworm, without impeding upon the spontaneous nature of the event. The experimenter asked each participant to test out both the tapping method and the button press in the laboratory. The experimenter then showed participants the paper diary and explained each question to ensure clarity.

Participants wore the accelerometer and carried the paper diary with them for a period of 4 days (96 hours). During this period, participants tapped to the beat of their earworms whenever possible and filled out the diary as soon as possible after the tapping period. They were debriefed as to the purposes of the experiment upon their return of the study materials.

### Analysis

#### Diary data analysis

Hand-written diary data was inputted into Microsoft Excel for further analysis in Excel and R. A total of 275 INMI episodes were reported in the diaries. Scores on each of the seven mood pairs were grouped into the three factors (Positivity, Arousal, and Present Mindedness) designated by the original authors of the mood scale (Sloboda et al., 2001) and summed. Scores on reverse-scored items were recalculated before adding as necessary. The Positivity factor comprised the happy/sad and tense/relaxed mood pairs, Arousal comprised the alert/drowsy and energetic/tired pairs, and Present Mindedness comprised the interested/bored, involved/detached, and lonely/connected pairs.

#### Tapping data analysis

To isolate individual tapping episodes, each participant’s movement data was viewed within the Data Analysis feature of the GeneActiv software. Each episode was located using the time and date reported in the diary booklet, with the button press as a marker of the episode endpoint. The start of a tapping episode was detected by examining the 2 min preceding the button press and locating the onset of a sequence of successive acceleration peaks corresponding to repetitive tapping. Once an episode was isolated, it was saved for further analysis. No discernable corresponding tapping sequence was found in the accelerometer data for ten of the episodes reported in the diaries (3.64 % of the reported episodes). This could be due to a variety of different reasons, such as the participant forgetting to tap, writing down the incorrect time in the diary, or not tapping a discernable beat pattern.

**Fig. 2:**
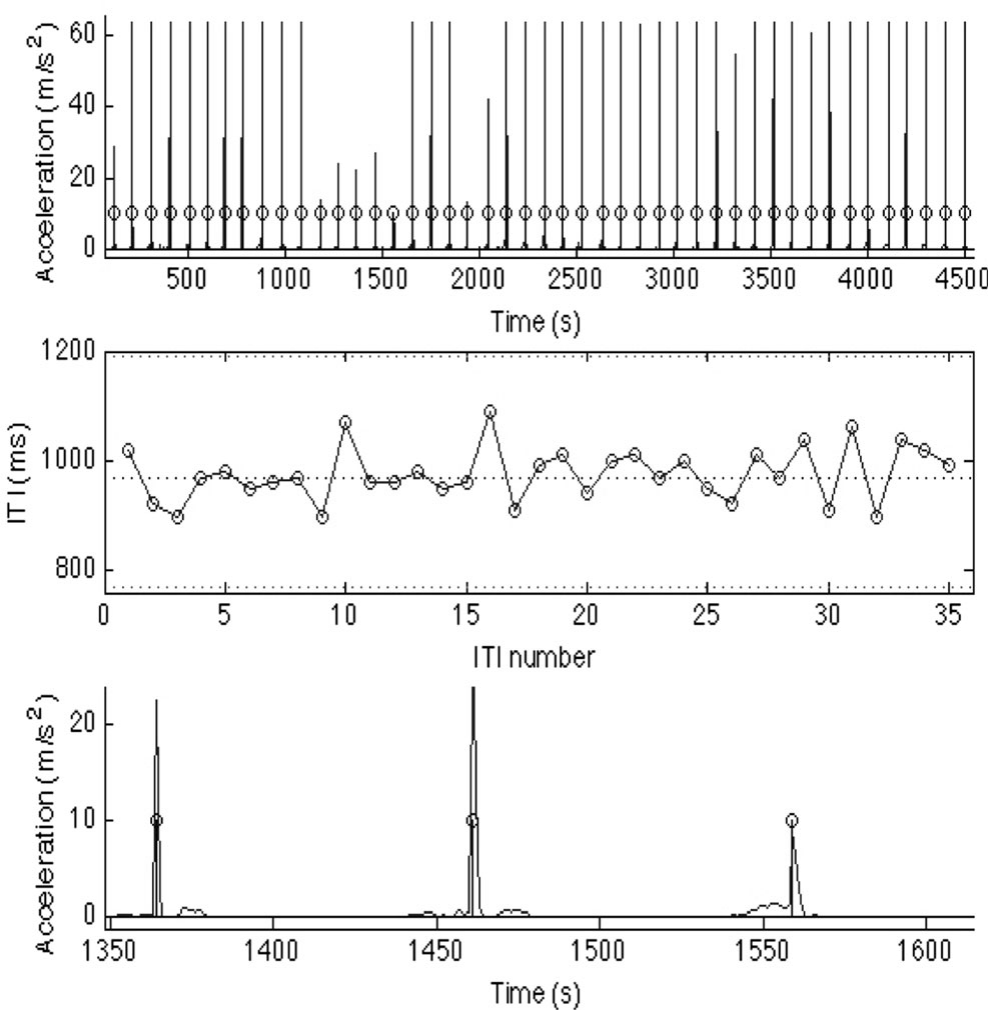
Graphical examples (from top to bottom) of (1) accelerometer movement data (minus the first ten excluded taps; circles denote local maxima), (2) series of corresponding inter-tap intervals, and (3) three individual taps from graph 1 (enlarged for clarity)

Next, each INMI episode was analyzed using a tap detection algorithm in MATLAB (see Fig. 2). The magnitude of the acceleration vector was computed as the square root of the sum of the three squared acceleration signals (x, y, and z). The resulting signal was smoothed using three passes of a running average filter in order to remove high frequency noise, and local maxima detection^5^ was performed on the smoothed signal. Detected maxima were considered as tap onsets if their absolute height was higher than a threshold; this threshold was defined as a ratio in relation to the highest maximum for the current tap sequence. The default threshold was set to 0.4, but was adjusted manually for each episode due to the fact that tapping strength and patterns varied greatly between and even within participants.

For each file, the resultant tap series was then processed using the following steps. The first ten taps were excluded from analysis, in line with previous tapping literature (e.g., Benoit et al., 2014; Sowiński & Dalla Bella, 2013; Zelaznik & Rosenbaum, 2010), and all numerical measurements were calculated based on the remaining taps. If there were fewer than ten remaining taps after excluding the first ten taps, this was recorded as a missing value, as the tapping period was deemed too short to extract a reliable tempo estimate. Overall, 30 INMI episodes were excluded on this basis (10.91 % of the total data).

Next, the time series of inter-tap intervals (ITI) was calculated as the difference between all successive tap onsets. This ITI series was further processed to remove artifacts and outliers (similar to the procedure used in Benoit et al., 2014). Artifacts occur when two taps are registered in brief succession, and can originate from rebounds (e.g., two fingers or two parts of the wrist/hand hit the tapping surface in brief succession) or signal glitches. In this case, artifacts were defined as ITI values of less than 100 ms. Outliers were defined as ITI values greater than three times the interquantile range from the median value of the ITI series, and usually represented missing taps. Overall, the average percentage of outliers (outliers divided by total number of taps) across all usable tapping sequences was 2.6 %, and 55.7 % of the usable tapping sequences contained no outliers at all. Using the artifact- and outlier-free ITI series, an average ITI value, coefficient of variation (CV; a normalized measure of tapping variability defined as the standard deviation of the ITI series divided by the mean ITI), and tempo in beats per minute (bpm) were outputted for each episode for further analysis.

Finally, all remaining tapping episodes were visually inspected in a graphical format in MATLAB (see Fig. 2). In this visual inspection stage, six episodes (2.18 % of the total data) were excluded on the basis of comprising a noisy signal without clearly discernable tapping peaks and one episode (0.36 % of the total data) was excluded on the basis of a participant halving the tempo in the middle of a tapping episode. Following these exclusion steps, 228 INMI episodes remained with usable tempo data (82.91 % of the total reported INMI episodes).

#### Mode data analysis

Two musicians were recruited to independently code the musical mode (i.e., major or minor) of each reported INMI episode. The coders followed a protocol resembling that of Schellenberg and von Scheve (2012), who also hand-coded the mode of pop songs. The coders were required to find a recording of each INMI tune, listen specifically to the section of the song reported as INMI by the participant, and code the mode as major, minor, or ambiguous.^6^ One of the present authors then collated the independent ratings of the two coders and examined any discrepancies. For 25 episodes, the participant provided insufficient information to determine the mode of the INMI tune. For the remaining 250 INMI episodes, the mode of 203 episodes (81.2 % of the remaining data) was coded identically by the two coders. Episodes that were not coded identically were excluded from further mode-related analyses on the basis of being tonally ambiguous. Of the 203 episodes that were coded identically, 160 were in major keys and 43 were in minor keys.

## Results

Descriptive statistics related to the music experienced as INMI and the circumstances surrounding the experience will be reported first to provide context and opportunities for comparison to previous literature. Results pertaining to the two main research questions of the paper —regarding the precision of tempo recall within INMI and the relationships between INMI and affective state—will then be reported.

### The INMI experience: Descriptive statistics

Of the 275 INMI episodes reported in the diaries, the number of episodes reported per participant during the full 4-day period ranged from seven to 32 episodes; the number of INMI episodes reported during one day ranged from zero to ten. The median number of episodes reported per participant was 16, or approximately four episodes per day (median episodes per day = 3.5). As reported in the *Tapping Data Analysis* section, a discernible corresponding tapping sequence was found in the accelerometer data for 265 of the 275 reported episodes. As the co-occurrence of both a tapping sequence and a diary entry provide strong evidence that an INMI experience actually occurred at the time it was reported, the following descriptive statistics are reported for these 265 episodes.

In total, 182 unique songs were reported as INMI. For the vast majority of episodes, the song title and performer were reported, indicating that the songs were familiar to participants; however, for 11 episodes both the title and performer field were left blank. Two participants reported experiencing self-composed music as INMI for one and two episodes respectively. Other reported songs comprised a mix of pop, classical, rock, rap, jazz, folk, musical theatre, Christmas, TV, and children’s music. Only one song (“Barbie Girl” by Aqua) was reported by two different participants; other repetitions of INMI songs occurred only within the same participant. A total of 42 songs were reported at least twice by the same participant, 15 songs were reported three or more times, and two songs were reported six times.

Participants also reported on how long it had been since they had heard the song experienced as INMI played aloud, e.g., on an iPod, radio, live performance, etc. For 16.2 % of episodes, the song experienced as INMI had been heard less than 1 hour ago, and for 23.4 % of episodes the song had been heard less than 3 hours ago. However, for 40.0 % of episodes, participants reported that they had not heard a recording or performance of the song experienced as INMI in over 1 week.

A total of 67 INMI episodes (25.3 % of episodes) were experienced during a repetitive movement. The majority of these (57 episodes) occurred while walking and three episodes occurred while typing. The remaining repetitive movements, which comprised a single report each, were: brushing teeth, climbing, cutting vegetables, cycling, dying hair, washing dishes, and washing hair.

**Fig. 3:**
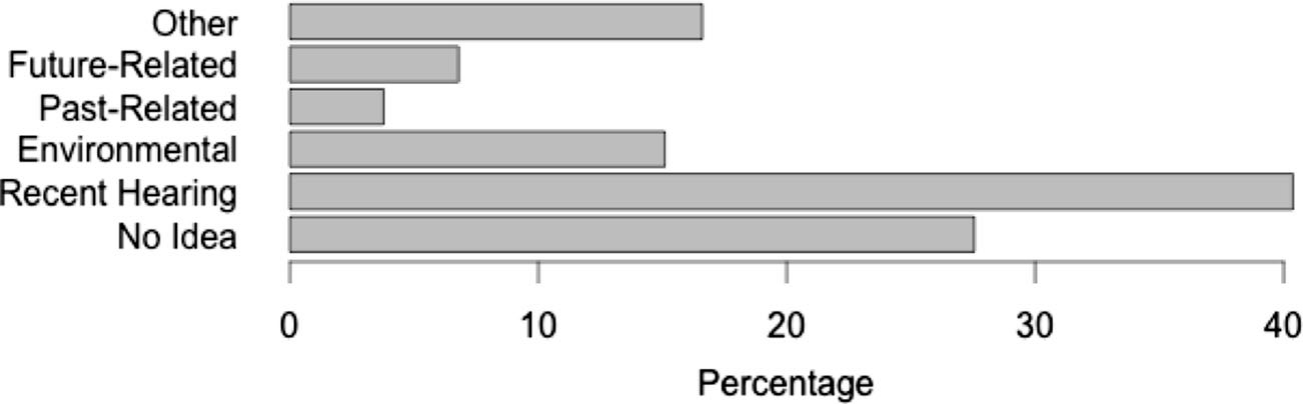
Percentages of involuntary musical imagery (INMI) episodes for which each trigger was reported. (Note: As multiple triggers could be reported for each episode, these percentages total to over 100 %)

Finally, participants were asked to report any reasons they thought a song might have occurred as INMI (see Fig. 3). Recent exposure to a song was named as a likely trigger for 40.4 % of INMI episodes. Association with an environmental trigger, such as a person, word, or sound was attributed as a potential cause in 15.1 % of episodes. For 27.6 % of episodes, participants reported, “I have no idea why this tune came into my head,” in the absence of any other trigger. Participants were also invited to record additional reasons an INMI episode might have occurred in response to an open question (if they ticked the box “Other”). “Other” reasons were reported for 16.6 % of episodes; however, a substantial proportion of these appeared to be instances where participants were providing additional details that still fit within existing categories. For instance, one participant wrote, “Aphex Twin is to release a new album on Sept. 22”, which could be classified in the category “I was thinking about a future event and related it to this song.” Additional recurring triggers reported in the “Other” category relate to features of a melody (N = 3; e.g., “it's a nice melody”) and mood states (N = 10; e.g., “maybe because I feel a bit tired, sitting at my desk makes me relax (it's a slow song)”).

### The tempo of INMI: Descriptive statistics

**Fig. 4:**
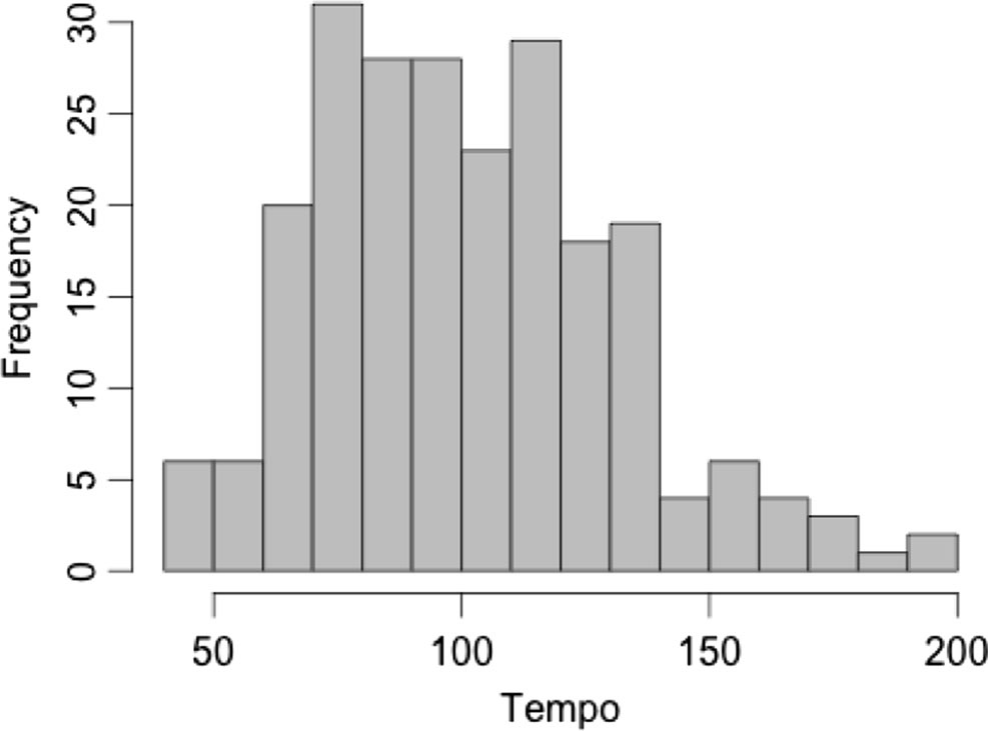
Tempo distribution of all 228 involuntary musical imagery (INMI) episodes with usable tempo data

Overall, 228 INMI episodes had usable tempo data (see *Tapping Data Analysis* section for data exclusion criteria). The number of total taps in each usable sequence ranged from 20 to 121 taps, and the duration of the tapping sequences ranged from 7.6 to 92.4 s (*M* = 23.6, *SD* = 10.1). These episodes ranged in tempo from 42.0 bpm to 196.5 bpm (see Fig. 4). The mean tempo across all INMI episodes was 100.9 bpm (*SD* = 29.9; median = 98.5). The mean CV (coefficient of variation) of tapping across all 228 episodes was 0.06 (*SD* = 0.02; range = 0.02–0.13).

#### Memory for INMI tempo: Veridicality and consistency

**Fig. 5:**
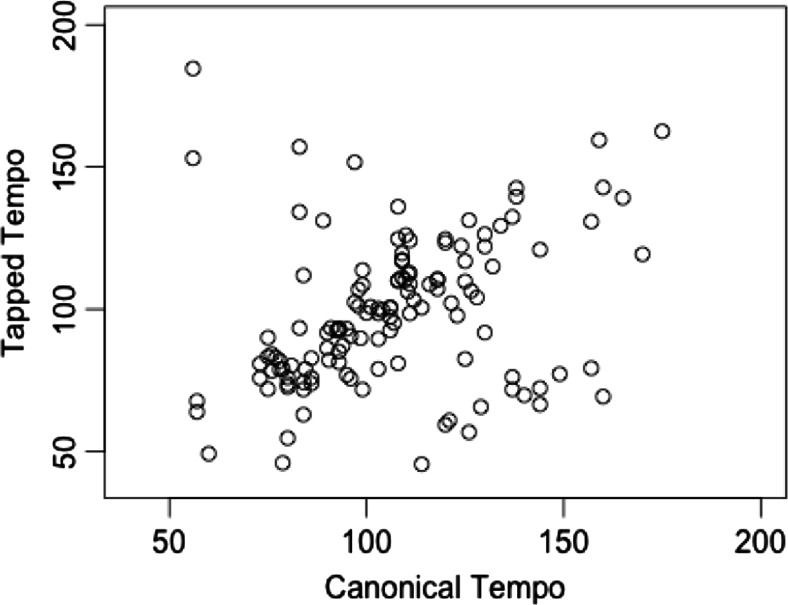
Original, recorded tempo for each of the 132 songs that exist in canonical versions plotted against the tempo each song was tapped at when experienced as involuntary musical imagery (INMI)

The next aim of the study was to investigate the veridicality of tempo recall within INMI, in comparison to the original version of each INMI tune. Of the 228 episodes with usable tempo data, 132 of these comprised INMI experiences of songs that exist in a canonical version. We define canonical songs as those that exist in one standard, recorded version. Examples of non-canonical songs include most Christmas songs and classical music, for which recordings might exist but no “definitive version” is apparent from which to obtain tempo information. The tapped tempo of each of these 132 INMI episodes was compared to the original, recorded tempo of the song by examining (1) ratios of the tapped to recorded tempo, (2) absolute deviations (as percentages) of the tapped tempo from the recorded tempo, and (3) the correlation between tapped and recorded tempo across all episodes (see Fig. 5). A ratio of 1 or an absolute deviation of 0 % for a particular episode would indicate that a participant tapped at the same tempo as the recorded version of the song.

Some extreme ratios of the tapped to recorded tempi likely represented participants halving or doubling the tempo of a song, i.e., tapping at a different metrical subdivision. In accordance with other previous research (Halpern, 1988; Jakubowski et al., 2014), episodes where the ratio of tapped to recorded tempo was 1.9 or greater or 0.6 or less were omitted, given the likelihood of participants having doubled or halved the song tempo. This resulted in the exclusion of 17 episodes, leaving 115 episodes for further analysis.

The mean ratio of tapped to recorded tempo for these 115 episodes was 0.98 (*SD* = 0.15, median = 0.97) and the mean absolute deviation of the tapped tempo from the original tempo was 10.8 % (*SD* = 10.8 %; median = 7.9 %). A highly significant correlation was also found between the tapped and original tempi, *r* = 0.77, *p* < .001. Overall, 59.1 % of songs were recalled within 10 % of the original recorded tempo and 77.4 % of songs were recalled within 15 % of the original tempo.

Next, it was investigated whether recency of hearing a song might influence INMI veridicality, such that songs heard aloud more recently might be recalled more accurately in terms of tempo. The data were split into two approximately equal subsets: songs heard within the past week (N = 64) and songs heard over one week ago (N = 51). A Wilcoxon rank-sum test was employed (due to non-normal distributions in the data) to compare these two subsets of the data in terms of their absolute deviations from the original tempo. The result of the test was non-significant, *W* = 1399, *p* = .19.

A final question into the veridicality of INMI was related to previous research in which Halpern (1988) reported a regression to the mean for the tempo of voluntarily imagined songs. The present data allowed for the first exploration into whether a regression to the mean might also be present for recalled tempo within INMI. An independent-samples *t*-test was performed to compare the original, recorded tempo between songs that were recalled slower than the recorded tempo within INMI (those songs with ratios of tapped tempo to recorded tempo of less than 1) and songs recalled faster than the recorded tempo (songs with ratios greater than 1). The mean recorded tempo for songs that were recalled slower than the original tempo was significantly faster than that of songs that were recalled faster than the original tempo, *t* (107) = 2.71, *p* = .01, thus suggesting regression to the mean within INMI.

Finally, the consistency of tempo recall for recurrences of the same tune as INMI was examined using the eight songs with usable tempo data that were experienced by the same participant at least three times. It should be noted that, unlike in the veridicality analyses, this sample of eight songs included both canonical and non-canonical songs. The mean tempo difference between the slowest and fastest version of a song experienced as INMI was 19.6 % (*SD* = 14.0 %, median = 14.6 %). When comparing the slowest and fastest rendition of each song, two of these eight songs differed in tempo by over 40 %. The remaining six songs differed in tempo by less than 20 %, and five songs differed by less than 15 % (see Table 1).

**Table 1:** Consistency of tempi for songs experienced as involuntary musical imagery (INMI) at least three times

Song	Number of INMI episodes	Slowest tempo (bpm)	Fastest tempo (bpm)	Difference between slowest and fastest version (%)
A Sky Full of Stars	3	82.5	116.9	41.7
Miss You	3	98.5	112.7	14.4
Ponta de Areia	3	63.0	74.3	18.0
Spirited Away One Summer's Day	3	78.6	82.6	5.1
One	4	81.4	93.2	14.4
You're So Vain	5	111.2	119.6	7.5
For Unto Us a Child is Born	6	74.6	105.0	40.6
Non Voglio Cantare	6	117.4	134.8	14.8

### Musical features of INMI and affective states

The second main research question investigated the relationship between participants’ affective states and specific musical features of their concurrent INMI. Specifically, (1) a positive relationship was predicted between the Arousal dimension of the mood scale and the tempo of INMI and (2) the Positivity dimension of participants’ mood ratings was predicted to be higher during INMI in the major versus the minor mode.

The correlations between INMI tempo, INMI mode, Arousal, and Positivity are reported in Table 2. Point-biserial correlations were calculated for the INMI mode variable due to its dichotomous nature; all other reported correlations are Pearson’s correlations.

**Table 2:** Correlations of musical features of involuntary musical imagery (INMI) and mood variables

	Tempo	Mode	Arousal	Positivity
Tempo	1.00			
Mode	–.003	1.00		
Arousal	.14*	.10	1.00	
Positivity	.15*	–.09	.07	1.00

*Note* * signifies a significant correlation at the level of *p* < .05. Coding for the INMI mode variable is: 1=minor, 2=major

A linear mixed-effect model was fitted with both Arousal and Positivity as predictors of INMI tempo. A mixed-effects model was employed in order to take account of the individual variations among participants and the multiple observations recorded from each participant by including “Participant” as a random effect in the model. In this model, Arousal was a significant positive predictor of INMI tempo and no significant relationship was found between Positivity and INMI tempo. The previously non-significant effect of Positivity was then removed and the model was refitted with only Arousal as a predictor of INMI tempo. Arousal was again a significant predictor and the reduced model provided a better model fit, based on the Bayesian Information Criterion (BIC), than the full model with both mood variables included as predictors (see Table 3). A pseudo-*R*^2^ value of 0.34 was obtained for the effect of arousal on INMI tempo by computing the squared the correlation between the INMI tempo values predicted from the mixed-effects model and the observed values of INMI tempo.

**Table 3:** Linear mixed-effects models with mood variables as predictors of *INMI* tempo

	Coefficient	S.E.	*t*-value	*p*-value
Model 1: Arousal and Positivity as predictors of INMI tempo				
Intercept	81.24	12.99	6.26	< .001
Arousal	1.87	0.67	2.78	.01*
Positivity	0.08	1.11	0.08	.94
Model 2: Arousal as a predictor of INMI tempo				
Intercept	82.06	7.23	11.35	< .001
Arousal	1.87	0.67	2.79	.01**

* signifies a significant predictor at the level of *p* < .05. BIC = 2163.98

** signifies a significant predictor at the level of *p* < .05. BIC = 2160.62

A second mixed-effects analysis was conducted with Arousal and Positivity as predictors of INMI mode. A binomial mixed-effects model was fit due to the binary nature of the INMI mode variable. Neither of the mood variables were significant predictors of INMI mode (see Table 4).

**Table 4:** Linear mixed-effects model with mood variables as predictors of INMI mode

	Coefficient	S.E.	*z*-value	*p*-value
Intercept	1.47	1.43	1.03	.30
Arousal	0.12	0.07	1.67	.10
Positivity	–0.10	0.13	–0.76	.45

## Discussion

The present study has contributed a number of novel results, demonstrating that the combination of naturalistic diary methods with a quantitative measurement device – in this case an accelerometer – can add a new dimension to research on ephemeral phenomena such as INMI. These data represent, to our knowledge, the first attempt towards an objective marker related to the occurrence and tempo of INMI during everyday life. Despite the lesser degree of experimental control over the research environment as compared to a laboratory setting, over 80 % of the acquired tapping data was usable for analysis. A wide range of INMI tempi, from approximately 40 to 200 bpm, provided a rich source of data that suggest a wide variety of personal inner music experiences.

### The precision of INMI tempo recall

INMI for music that exists in a canonical version was generally experienced at a tempo very close to the veridical tempo of the song. In previous research on absolute memory for musical tempo, Levitin & Cook (1996) asked participants to sing self-selected, familiar pop songs and reported that 72 % of trials were within 8 % of the original, recorded tempo. Jakubowski et al. (2014) asked participants to deliberately imagine and tap to the beat of familiar pop songs and reported a mean absolute deviation from the original recorded tempo of 17.3 %. In the present study, the mean absolute deviation from the original tempo for INMI episodes was 10.8 % and 77.4 % of songs were recalled within 15 % of the original tempo. These figures suggest that tempo representations within INMI may be equally or even more veridical than those within musical imagery that is deliberately imagined in a laboratory context. Both forms of imagery appear to be less temporally veridical than songs produced in a sung recall paradigm (Levitin & Cook, 1996). However, as Levitin and Cook asked participants to sing only two self-selected songs that they knew very well, familiarity or overlearning may have played a role in the higher level of veridical recall observed in their study. Veridicality of tempo recall within the present study may also have been influenced negatively if participants had heard other versions of the songs they reported as INMI. However, as participants were asked to report the performer of the version of the tune they were experiencing as INMI, cover versions should have been reported as such.

Overall, the finding of high temporal veridicality within INMI is particularly striking given that (1) the songs reported as INMI were recalled spontaneously with no instruction for veridical recall^7^ and (2) the INMI occurred within the context of the external distractions of everyday life. The high veridicality of tempo within INMI suggests a parallel between involuntarily and voluntarily recalled musical memories. This finding also suggests a parallel to involuntary autobiographical memories, which have a tendency to be even *more* specific and detailed than voluntary autobiographical memories (Berntsen, 1998; Mace, 2006; Schlagman & Kvavilashvili, 2008). Future studies that directly compare involuntarily and voluntarily generated musical memories within the same participants could shed further light on whether INMI may also be more veridical or vivid in some cases than voluntary musical imagery.

In 40 % of INMI episodes in the present study, participants reported that they had not heard the song that was experienced as INMI in over one week. However, INMI for these songs was not experienced at a less veridical tempo than INMI for songs that had been heard more recently. This finding suggests that INMI that are recalled from long-term memory are temporally precise, and that the overall finding of high veridicality for INMI tempo is not explained solely by those episodes for which an INMI song was heard minutes ago on the radio and might still be held in short-term memory.

Evidence was also found for a regression to the mean for INMI tempo, such that faster songs tended to be recalled slower than their original tempi and slower songs tended to be recalled faster than their original tempi. This parallels previous findings on tempo for voluntary musical imagery (Halpern, 1988). Further research should be conducted to explore the mechanisms underlying this regression toward a mid-range tempo in both spontaneous and deliberate musical imagery, and whether this tendency is related to factors such as one’s natural, spontaneous tapping rate or preferred perceptual tempo (e.g., McAuley et al., 2006).

An exploratory analysis was conducted to examine the temporal consistency for the eight INMI tunes with usable tempo data that were reported at least three times. The majority of these INMI tunes differed in tempo between the slowest and fastest rendition by less than 20 %, however two songs varied in tempo by approximately 40 %. For one of these less consistent songs (“For Unto Us a Child is Born”), the participant who reported this song stated in the post-experiment debrief session that he was a pianist who often practiced musical pieces at different tempi when learning them, and that this seemed to influence his subsequent INMI tempi. This is just one example of a variety of reasons that might affect the consistency of INMI tempi that could not be accounted for in the present study. In future research, it may be fruitful to employ a design that aims specifically to examine the issue of temporal consistency. Such a design might involve priming participants with a recording of a song for which only one version exists and asking participants to record the tempo of all subsequent INMI episodes related to that particular song. Controlling the exposure phase of the INMI tune would consequently provide more control over the version of the song that comes to participants’ minds as INMI.

One potential limitation of the present research design is that, as the study was completed outside of the experimenter’s supervision, participants could not be prevented from voluntarily manipulating the tempo of their INMI. However, the present study aimed to combat this issue in several ways: (1) by providing a clear definition of “earworms” to participants that emphasized their involuntary recall as a key feature, (2) by not requiring any sort of button press or marker *before* the tapping period, so as to make it easy to start tapping as soon as possible when an INMI episode began, (3) by not revealing the purposes of the study until after it was completed, and (4) by instructing participants specifically to “please tap the beat of the tune as closely as possible as to what you hear in your head.” This last point is similar to the instructions utilized by Ericsson and Simon (1993) in the “think-aloud” method, which aims to capture participants’ current thoughts from within working memory, rather than their interpretation or justification of these thoughts.

### The relationship between INMI and affective states

Examining the relationships between musical features of INMI and concurrent affective states allowed the present research to begin to unravel the complex question of how endogenous bodily and mental states may interact with spontaneous cognitions. The present results revealed a modest yet significant positive relationship between subjective arousal and INMI tempo. This parallels and strengthens findings on the relationship between musical tempo and arousal in the domain of music listening (Edworthy & Waring, 2006; Husain et al., 2002; North & Hargreaves, 2000) by demonstrating that even music that is experienced only as imagery (i.e., not heard aloud) *and* spontaneously generated displays this significant tempo-arousal relationship. It is not possible to deduce from these results a direction of causality of this effect. However, previous studies of perceived music suggest a bidirectional relationship, such that tempo can influence one’s arousal level (e.g., Husain et al., 2002) and arousal can influence one’s tempo preferences (North & Hargreaves, 2000) or the tempo at which a piece of music sounds “right” (Jakubowski et al., 2015). As such, a similar bidirectional relationship might exist between musical tempo recalled with INMI and concurrent arousal.

No significant relationship was found between INMI mode and positivity of mood. This was unexpected, given previous evidence of an association between emotional valence and musical mode (Gagnon & Peretz, 2003; Husain et al., 2002; Webster & Weir, 2005). One potential explanation is that musical mode may be a less prominent or distinctive feature of INMI in comparison to other musical features of the imagery. INMI tempo might be a more salient feature of the imagery than musical mode because INMI tempo is more likely to influence motor responses or, conversely, to be influenced by movements that were occurring before the INMI episode began, thus making it more observable to one’s conscious awareness. This idea is supported by the evidence of a close relationship between musical beat perception and motor areas of the brain (Grahn & Brett, 2007; Grahn & Rowe, 2009), as well as by the finding that approximately one quarter of INMI occurred during a repetitive movement in the present study. The study instructions, in which participants were asked to move to the beat of the music by tapping, may have also increased the salience of INMI tempo over other musical features.

The relationship between INMI tempo and subjective arousal suggests some first evidence that INMI might be functionally linked to mood regulation in a similar manner to perceived music. That is, in the absence of an iPod or other music-generating device, spontaneous imagery for music may be able to fill in as a mood regulatory mechanism. Further research should be conducted to examine other musical dimensions of INMI (e.g., lyrical content, timbre, loudness) in order to gain a more complete understanding of the possible relationships between different features of the music experienced as INMI and concurrent mood.

### The INMI experience: Descriptive findings

Finally, several descriptive findings from the diary data corroborated and extended previous research. The diverse variety of songs reported (182 in total) and the lack of overlap in INMI tunes between participants is in line with several previous studies, suggesting that almost any song can become INMI and that the INMI experience is highly personal and idiosyncratic (Beaman & Williams, 2010; Williamson & Müllensiefen, 2012). The phenomenon has also been found to be short-lived (Byron & Fowles, 2013), as affirmed by the relatively few songs reported three or more times as INMI within each participant.

The present study also served as one of the first diary-based investigations into the triggers of the INMI experience. Recent exposure to music was the most commonly reported INMI trigger, which is in line with a retrospective questionnaire-based method employed by Williamson et al. (2012). However, it should be noted that, as the present study also included a question about how recently a participant had heard a song aloud, participants might have been somewhat primed towards reporting recent exposure to a song as an INMI trigger. The study also revealed that conscious awareness of an external or internal trigger for the experience was absent in about one quarter of INMI episodes, those in which a participant reported, “I have no idea why this tune came into my head.” Additionally, ten reports of an INMI episode being triggered in relation to a mood state were collected. In subsequent larger-scale studies, an investigation could be conducted into whether these specifically mood-triggered INMI display a stronger relationship between mood ratings and the musical features of INMI than INMI activated by other sources.

Finally, data on concurrent movements during INMI indicated that approximately one quarter of INMI episodes occurred during a repetitive movement, such as walking. These data provide some impetus for future accelerometer-based research, in which one could investigate how concurrent movement and INMI tempo might influence one another, e.g., if INMI tempo changes to match a movement or if a movement becomes more regular when it is made in time with INMI, by comparing acceleration patterns before and during INMI. Such research could provide valuable insights into the interactions between musical imagery and the sensorimotor system.

## Conclusion

The present paper has introduced a novel methodology that opens new avenues for research into dynamic aspects of spontaneous and self-generated thoughts. INMI is an example of a spontaneous cognition with several highly measurable features. The present study specifically investigated tempo-related aspects of the INMI experience within the course of everyday life.

The results of the study demonstrated that INMI is often a highly veridical experience in terms of tempo, even when a song experienced as INMI has not recently been heard aloud. The results also revealed a significant positive relationship between subjective arousal and INMI tempo, suggesting a first link between spontaneous musical imagery and mood that parallels findings in the perceived music domain. Research into temporal aspects of INMI and involuntary memories in general opens an array of important questions that will help further our understanding of endogenous thought processes.

## Footnotes

1 Although the term *involuntary musical imagery* (*INMI*) is used throughout this paper as a more complete description of the phenomenon, the term *earworm* was used in all instructions given to participants, as this represents a more familiar and colloquial term. Hence, the term *earworm* will be used in this paper only when describing the instructions given to participants.

2 Outliers were defined as data points more than 1.5 interquartile ranges below the first quartile or above the third quartile.

3 http://www.geneactiv.org/

4 Tap onset times were recorded by detecting a change in the status of the touchpad using the output of the fingerpinger object for MAX (available at http://www.anyma.ch/2009/research/multitouch-external-for-maxmsp/).

5 Both of these steps were implemented using the lmax function, available at http://www.mathworks.com/matlabcentral/fileexchange/3170-local-min-max-nearest-neighbour/content/lmax.m.

6 In the case of songs with no definitive version, such as classical music and Christmas songs, the coders were requested to find one standard recording, as it was not presumed that mode would change between different recorded versions of the song.

7 This is in contrast to both laboratory studies reported above (Jakubowski et al., 2014; Levitin & Cook, 1996), who asked participants to recall each song as closely as possible to the original version.
